# Characteristics and Treatments of Patients Enrolled in the CHAMP‐HF Registry Compared With Patients Enrolled in the PARADIGM‐HF Trial

**DOI:** 10.1161/JAHA.118.009237

**Published:** 2018-06-12

**Authors:** Adam D. DeVore, Xiaojuan Mi, Laine Thomas, Puza P. Sharma, Nancy M. Albert, Javed Butler, Adrian F. Hernandez, J. Herbert Patterson, John A. Spertus, Fredonia B. Williams, Carol I. Duffy, Kevin McCague, Gregg C. Fonarow

**Affiliations:** ^1^ Duke Clinical Research Institute Durham NC; ^2^ Department of Medicine Duke University School of Medicine Durham NC; ^3^ Cleveland Clinic Cleveland OH; ^4^ University of Mississippi Medical Center Jackson MS; ^5^ Eshelman School of Pharmacy University of North Carolina Chapel Hill NC; ^6^ Saint Luke's Mid America Heart Institute and the University of Missouri–Kansas City Kansas City MO; ^7^ Mended Hearts Huntsville AL; ^8^ Novartis Pharmaceuticals Corporation East Hanover NJ; ^9^ Ahmanson‐UCLA Cardiomyopathy Center Ronald Reagan UCLA Medical Center Los Angeles CA

**Keywords:** angiotensin receptor neprilysin inhibitor, quality improvement, registry, sacubitril/valsartan, Heart Failure, Quality and Outcomes

## Abstract

**Background:**

The US Food and Drug Administration approved sacubitril/valsartan for patients with chronic heart failure (HF) with reduced ejection fraction in 2015 on the basis of the results of the PARADIGM‐HF (Prospective Comparison of ARNI [Angiotensin Receptor Neprilysin Inhibitor] With ACEI [Angiotensin‐Converting Enzyme Inhibitor] to Determine Impact on Global Mortality and Morbidity in Heart Failure) trial. There are limited data assessing the generalizability of PARADIGM‐HF trial participants to a broader population of patients with HF with reduced ejection fraction routinely encountered in outpatient clinical practice.

**Methods and Results:**

We compared the baseline characteristics of patients in the PARADIGM‐HF trial with those in the CHAMP‐HF (Change the Management of Patients With Heart Failure) study a large US outpatient registry of patients with HF with reduced ejection fraction. Patients in the PARADIGM‐HF trial (n=8442) were similar to those in the CHAMP‐HF registry (n=3497) in terms of age (mean, 64 versus 66 years), sex (22% versus 29% women), New York Heart Association class III to IV (25% versus 32%), systolic blood pressure (mean, 121 versus 121 mm Hg), left ventricular ejection fraction (mean, 29% versus 29%), and other key baseline characteristics. The median (25th–75^th^ percentile) Meta‐Analysis Global Group in Chronic Heart Failure risk scores were similar for the 2 studies (20 [16–24] versus 22 [8–27]). Despite this, only 13% of patients in the CHAMP‐HF registry were prescribed sacubitril/valsartan at baseline.

**Conclusions:**

These data suggest participants randomized in the PARADIGM‐HF trial have similar baseline characteristics to those encountered in routine outpatient clinical practice, but there is a substantial lag in the adoption of sacubitril/valsartan for patients with chronic HF with reduced ejection fraction.

**Clinical Perspective**

**What Is New?**

- We compared the baseline characteristics of outpatients with heart failure with reduced ejection fraction enrolled in a contemporary US registry with the baseline characteristics of patients randomized in the PARADIGM‐HF (Prospective Comparison of ARNI [Angiotensin Receptor Neprilysin Inhibitor] With ACEI [Angiotensin‐Converting Enzyme Inhibitor] to Determine Impact on Global Mortality and Morbidity in Heart Failure) trial.

**What Are the Clinical Implications?**

- We found similar baseline characteristics and risk of all‐cause mortality in the 2 populations, suggesting that the population of patients with heart failure enrolled in the PARADIGM‐HF trial largely reflects patients with heart failure encountered in outpatient clinical practice.

## Introduction

Current heart failure (HF) guidelines recommend the use of an angiotensin receptor neprilysin inhibitor in a broad population of patients with chronic HF with reduced ejection fraction (HFrEF).^1^ The guidelines recommend an angiotensin receptor neprilysin inhibitor, an angiotensin‐converting enzyme inhibitor (ACEI), or an angiotensin receptor blocker (ARB) to reduce morbidity and mortality in all eligible patients with stage C chronic HFrEF. In addition, the guidelines recommend, in patients with New York Heart Association (NYHA) class II to III symptoms who can tolerate an ACEI or ARB, that an angiotensin receptor neprilysin inhibitor replace the ACEI or ARB to further reduce morbidity and mortality. Both of these recommendations are class I, level of evidence B‐R on the basis of the results of the PARADIGM‐HF (Prospective Comparison of ARNI [Angiotensin Receptor Neprilysin Inhibitor] With ACEI to Determine Impact on Global Mortality and Morbidity in Heart Failure) trial.^2^

The specific inclusion and exclusion criteria of the PARADIGM‐HF trial (eg, patients with history of symptomatic hypotension or systolic blood pressure [SBP] <95 mm Hg at randomization were excluded) and the sequential active run‐in period have led to concerns that PARADIGM‐HF trial participants were a highly select population of patients with chronic HFrEF.^3^ To understand how patients randomized in the PARADIGM‐HF trial may or may not be reflective of those routinely encountered in current clinical practice, we compared the baseline characteristics of patients in the PARADIGM‐HF trial with those in the CHAMP‐HF (Change the Management of Patients With Heart Failure) study a large US outpatient registry of patients with HFrEF with limited inclusion and exclusion criteria.

## Methods

The data, analytical methods, and study materials will not be made available to other researchers for purposes of reproducing the results or replicating the procedure. The study design of the CHAMP‐HF registry was previously published.^4^ In brief, the CHAMP‐HF registry is a prospective observational study of outpatients with chronic HFrEF (left ventricular ejection fraction [LVEF], ≤40%).^4^ To be eligible, all participants must be receiving treatment with at least 1 oral pharmacotherapy for management of HF. The participants are being recruited and followed up as part of routine outpatient HF management at 152 sites across the United States. The registry is a prospective, observational, cohort study without intervention, and there was no attempt to influence clinical practice. This study is ongoing, and the baseline characteristics have not been previously published. All patients are required to sign written informed consent before collection of any study data. The CHAMP‐HF registry is sponsored by Novartis Pharmaceuticals Corporation (East Hanover, NJ). Data are managed by the United BioSource Corporation (Blue Bell, PA), and the Duke Clinical Research Institute (Durham, NC) is the data analytic center. Eligibility criteria of the CHAMP‐HF and PARADIGM‐HF studies are compared in Table 1. The baseline characteristics of PARADIGM‐HF trial patients were previously published.^5^, ^6^

**Table 1:** Key Enrollment Criteria for the PARADIGM‐HF and CHAMP‐HF Studies

Variable	PARADIGM‐HF (N=8442)	CHAMP‐HF (N=5000)
Recruitment time period	2009–2012	2015–2017
Key enrollment criteria		
Age, y	≥18	≥18
NYHA class	II–IV	No restriction specified
LVEF, %	≤40^a^	≤40
Prior HF hospitalization	Yes^b^	No restriction specified
BNP or NT‐proBNP, pg/mL	BNP ≥150 or NT‐proBNP ≥600^b^	No restriction specified
eGFR, mL/min per 1.73 m^2^	≥30	No restriction specified
Systolic BP, mm Hg	≥95	No restriction specified
Potassium, mmol/L	≤5.4	…
Prior medical therapy	ACEI (enalapril, 10 mg/d) or equivalent for 4 wk β Blocker for 4 wk MRA considered	At least 1 oral pharmacotherapy for HF
Run‐in period with active treatment	Yes	Not applicable

ACEI indicates angiotensin‐converting enzyme inhibitor; BNP, B‐type natriuretic peptide; BP, blood pressure; CHAMP‐HF, Change the Management of Patients With Heart Failure; eGFR, estimated glomerular filtration rate; HF, heart failure; LVEF, left ventricular ejection fraction; MRA, mineralocorticoid receptor antagonist; NT‐proBNP, N‐terminal pro‐B‐type natriuretic peptide; NYHA, New York Heart Association; PARADIGM‐HF, Prospective Comparison of ARNI [Angiotensin Receptor Neprilysin Inhibitor] With ACEI to Determine Impact on Global Mortality and Morbidity in Heart Failure.

a Initially, the required LVEF was ≤40%, but this was changed to ≤35% in a protocol amendment in December 2010.

b Plasma BNP ≥150 pg/mL (or NT‐proBNP ≥600 pg/mL) at the screening visit or a BNP ≥100 pg/mL (or NT‐proBNP ≥400 pg/mL) with a hospitalization for HF within the past 12 months.

The current study includes CHAMP‐HF registry patients with baseline visits performed between December 14, 2015, and March 6, 2017. Patients with missing baseline demographics were excluded (n=21 [0.60%]). We described baseline characteristics of the study population using proportions for categorical variables and means with SDs for continuous variables. The baseline characteristics were not compared using traditional statistical probability testing. Because of the sample size, we were concerned differences may by statistically different, although they may not have clinically meaningful differences. For baseline characteristics, most variables had few to no missing values (<0.5% missing); notable exceptions included 8% for body mass index, 5% for SBP, 4% for NYHA class, <1% for LVEF, and 7% for pulse. Renal function was also missing at a higher rate (32% for creatinine) because the CHAMP‐HF registry only includes laboratory information captured as part of routine care. Descriptive data were reported on complete cases (ie, nonmissing).

The Meta‐Analysis Global Group in Chronic Heart Failure (MAGGIC) risk score for mortality in HF was reported as a median with an interquartile range.^7^ Variables required for calculation of the MAGGIC risk score include age, sex, body mass index, current smoker, SBP, diabetes mellitus, NYHA class, LVEF, chronic obstructive pulmonary disease, HF duration, serum creatinine, and medication use. To calculate the MAGGIC risk score, we applied single imputation, using a fully conditional specification, for missing values for body mass index, SBP, NYHA class, LVEF, and creatinine. All analyses were performed using SAS software, version 9.4 (SAS Institute, Cary, NC).

## Results

The study population at the time of the current analysis included 3497 patients from 140 sites. Table 2 shows the baseline characteristics of the PARADIGM‐HF trial participants as well as the CHAMP‐HF registry population. Patients randomized in the PARADIGM‐HF trial (n=8442) were similar to those enrolled in the CHAMP‐HF registry in terms of age (64 versus 66 years), sex (22% versus 29% women), NYHA class III to IV (25% versus 32%), SBP (mean, 121 versus 121 mm Hg), diastolic blood pressure (mean, 74 versus 73 mm Hg), estimated glomerular filtration rate (mean, 68 versus 60 mL/min per 1.73 m^2^), and LVEF (mean, 29% versus 29%). Patients enrolled in the PARADIGM‐HF trial had lower rates of hypertension (71% versus 82%), higher rates of an ischemic cause for their HF (60% versus 40%), and lower rates of implantable cardioverter defibrillator use (15% versus 42%). The median (25th–75^th^ percentile) MAGGIC risk scores were similar for the 2 studies (PARADIGM‐HF, 20 [16–24]; CHAMP‐HF, 22 [8–27]). Of 3497 patients enrolled in the CHAMP‐HF registry, 452 (13%) were prescribed sacubitril/valsartan, 37 (1.1%) had a documented contraindication to sacubitril/valsartan, and 190 (5.4%) had a documented contraindication to ACEI and/or ARB. Patients enrolled in the CHAMP‐HF registry had the following forms of insurance: Medicare (58%), managed care (16%), private (9.4%), Medicaid (9.1%), and other/uninsured (6.7%).

**Table 2:** Baseline Characteristics and Treatments in the CHAMP‐HF and PARADIGM‐HF Studies

Variable	PARADIGM‐HF (N=8442)	CHAMP‐HF (N=3497)
Age, mean (SD), y	64	66 (13)
Female sex, %	22	29
Race, %		
White	66	75
Black	5	16
Asian	18	2
Other	11	7
NYHA class		
I	5	10
II	70	55
III	24	29
IV	1	3
Heart rate, mean (SD), bpm	72	74 (12)
Blood pressure, mean (SD), mm Hg		
Systolic	121	121 (18)
Diastolic	74	73 (11)
LVEF, mean (SD), %	29	29 (8)
BMI, mean (SD), kg/m^2^	28	30 (7)
Medical history, %		
Ischemic cause	60	40
HF hospitalization^a^	63	38
Hypertension	71	82
Angina pectoris	27	11
Myocardial infarction	43	23
PCI	21	24
CABG	15	18
Atrial fibrillation	37	36
Diabetes mellitus	34	41
Stroke	9	9
Tobacco use within 12 mo	14	20
Renal function		
Creatinine, mean (SD), μmol/L^b^	99	108 (43)
eGFR, mean (SD), mL/min per 1.73 m^2^	68	60 (20)
eGFR <60 mL/min per 1.73 m^2^, %	37	26
Treatment, %		
Loop diuretic	80	61
ACEI	77	41
ARB	22	20
ACEI, ARB, or both	100^c^	60
ARNI	···	13
β Blocker	93	83
MRA	60	33
Digoxin	30	14
Anticoagulant	32	26
Aspirin	52	57
ADP antagonist	15	24
Any antiplatelet	57	63
Lipid‐lowering agent	56	67
CRT	7	7
ICD	15	42
MAGGIC risk score, median	20	22

ACEI indicates angiotensin‐converting enzyme inhibitor; ADP, adenosine diphosphate receptor; ARB, angiotensin receptor blocker; ARNI, angiotensin receptor neprilysin inhibitor; BMI, body mass index; bpm, beats per minute; CABG, coronary artery bypass grafting; CHAMP‐HF, Change the Management of Patients With Heart Failure; CRT, cardiac resynchronization therapy; eGFR, estimated glomerular filtration rate; HF, heart failure; ICD, implantable cardioverter‐defibrillator; LVEF, left ventricular ejection fraction; MAGGIC, Meta‐Analysis Global Group in Chronic Heart Failure; MRA, mineralocorticoid receptor antagonist; NYHA, New York Heart Association; PARADIGM‐HF, Prospective Comparison of ARNI With ACEI to Determine Impact on Global Mortality and Morbidity in Heart Failure; PCI, percutaneous coronary intervention; SD, standard deviation.

a In the CHAMP‐HF registry, HF hospitalizations were considered if within the previous 12 months, whereas in the PARADIGM‐HF trial, there was no time limit.

b SDs only available for the CHAMP‐HF registry cohort.

c To be eligible for the PARADIGM‐HF trial, patients had to be treated with a stable dose of an ACEI or ARB equivalent of enalapril, 10 mg/d.

## Discussion

In this study, we describe the baseline characteristics of outpatients with HFrEF enrolled in the CHAMP‐HF registry and compared them with patients randomized in the PARADIGM‐HF trial. We found that despite different inclusion/exclusion criteria for the 2 studies, patients enrolled in the PARADIGM‐HF trial had similar baseline characteristics to those enrolled in the CHAMP‐HF registry. They also had similar projected risk of all‐cause mortality, as assessed by the MAGGIC risk score, where a score of 20 (PARADIGM‐HF) is associated with a 1‐year risk of death of 10.2% and a score of 22 (CHAMP‐HF) is associated with a 1‐year risk of death of 12.2%.^7^ These findings suggest that the population of patients with HFrEF enrolled in the PARADIGM‐HF trial largely reflects patients with HFrEF encountered in outpatient clinical practice. The use of sacubitril/valsartan, however, was only 13% at baseline in the CHAMP‐HF registry and highlights an important treatment gap in the implementation of recent HF guideline recommendations.

A prior study applied the eligibility criteria of the PARADIGM‐HF trial to 210 patients hospitalized with acute or chronic HFrEF at the Cleveland Clinic with available follow‐up data within 30 days of discharge.^3^ Of these 210 patients, 71% were eligible for sacubitril/valsartan at the follow‐up appointment on the basis of Food and Drug Administration labeling criteria, yet only 26% were eligible for enrollment in the PARADIGM‐HF trial on the basis of the trial eligibility criteria. The primary reasons patients would not have been eligible for the PARADIGM‐HF trial were low SBP, impaired renal function, and elevated potassium. The authors highlighted the difference in criteria for Food and Drug Administration labeling and enrollment in the PARADIGM‐HF trial and concluded that the PARADIGM‐HF trial population represented a minority of patients with HFrEF. The proportion of patients eligible for enrollment in the PARADIGM‐HF trial in the Cleveland Clinic study was lower than what was observed (38%) in a similar analysis of patients from Get With The Guidelines‐HF.^8^ Our current study differs in that we studied a broad population of outpatients with chronic HFrEF, not those recently hospitalized with acute HF, and we compared baseline characteristics in lieu of applying trial eligibility criteria.

Although our current study population is similar to that of the PARADIGM‐HF trial, our data do not alter the need for additional data on the safety and efficacy of sacubitril/valsartan in different populations of patients with HFrEF, including hospitalized patients, those with NYHA class I and IV HF, and other populations not well represented in the PARADIGM‐HF trial. Ongoing clinical trials, including the PIONEER‐HF (Comparison of Sacubitril/Valsartan Versus Enalapril on Effect on NT‐Pro‐BNP in Patients Stabilized From an Acute Heart Failure Episode) study^9^ and the TRANSITION (Comparison of Pre‐ and Post‐Discharge Initiation of LCZ696 Therapy in HFrEF Patients After an Acute Decompensation Event) study,^10^ will provide valuable information about the efficacy and safety of sacubitril/valsartan in patients with a recent episode of acute HF.

The adoption of sacubitril/valsartan into routine clinical practice was low early after Food and Drug Administration approval, as evidenced by our study as well as other studies.^11^ There were likely multiple reasons for this, including patient and provider concerns about hypotension, generalizability of trial results, and cost given that ACEIs and ARBs are available as generic medications. However, this pattern of adoption is not unique to sacubitril/valsartan, as described in a prior Institute of Medicine report noting an average of 17 years for new knowledge generated by randomized clinical trials to be incorporated into practice.^12^ The slow early adoption of sacubitril/valsartan underscores the need for robust evidence on best implementation strategies, as noted in a recent special report by the National Heart, Lung, and Blood Institute Implementation Science Work Group.^13^ This is being addressed in an ongoing cluster randomized trial of quality improvement strategies for patients hospitalized with HFrEF, CONNECT‐HF (Care Optimization Through Patient and Hospital Engagement Clinical Trial for Heart Failure; NCT03035474).

Our study has limitations. Participation in the CHAMP‐HF registry involves voluntary participating sites and requires signed informed consent and the ability to complete multiple surveys, and this may select for a specific population of patients with HFrEF. Also, the CHAMP‐HF registry is an ongoing study. We present data on only 3497 patients compared with 8000 patients in the PARADIGM‐HF trial, and the adoption of sacubitril/valsartan is improving over time. For example, the proportion of patients prescribed sacubitril/valsartan at enrollment in the registry increased from 9% to 14% during the study period.

## Conclusions

In the CHAMP‐HF study a large registry of outpatient practices in the United States, baseline characteristics and risk of mortality were similar to participants randomized in the PARADIGM‐HF trial, reflecting generalizability of PARADIGM‐HF trial participants to a broader population of patients with HFrEF routinely encountered in outpatient clinical practice. The adoption of sacubitril/valsartan remains low, underscoring the need for better implementation strategies for patients with chronic HFrEF to more efficiently translate new knowledge into practice.

## Sources of Funding

The CHAMP‐HF (Change the Management of Patients With Heart Failure) registry is sponsored by Novartis Pharmaceuticals Corporation.

## Disclosures

DeVore reports research funding from the American Heart Association, Amgen, the National Heart, Lung, and Blood Institute, and Novartis and serving as a consultant for Novartis. Thomas reports research funding from Novartis. Albert reports serving as a consultant to Novartis and Boston Scientific. Butler has received research support from the National Institutes of Health (NIH), Patient‐Centered Outcomes Research Institute (PCORI), and the European Union and serves as a consultant for Amgen, Array, Astra Zeneca, Bayer, Boehringer Ingelheim, Bristol Myers Squib, CVRx, G3 Pharmacautical, Innolife, Janssen, Luitpold, Medtronic, Merck, Novartis, Relypsa, StealthPeptide, SC Pharma, Vifor, and ZS Pharma. Hernandez has received research support from the American Heart Association, AstraZeneca, Merck, the National Heart, Lung, and Blood Institute, Luitpold, and Novartis and honorarium from Bayer, Boston Scientific, and Novartis. Patterson reports research funding from Amgen, Bristol‐Myers Squibb, Merck, and Novartis and serves as a consultant to Amgen and Novartis. Spertus reports research funding from NIH, PCORI, Abbott Vascular, and Lilly. He serves as a consultant to Novartis, Bayer, and United Healthcare. He owns the copyright to the Seattle Angina Questionnaire, Kansas City Cardiomyopathy Questionnaire, and Peripheral Artery Questionnaire. He has an equity interest in Health Outcomes Sciences. Sharma, Duffy, and McCague report employment by Novartis Pharmaceuticals Corporation (East Hanover, NJ). Fonarow reports research funding from the NIH and consulting for Amgen, Janssen, Novartis, Medtronic, and St Jude Medical. The remaining authors have no disclosures to report.
